# The Impact of Suicide Bereavement on Educational and Occupational Functioning: A Qualitative Study of 460 Bereaved Adults

**DOI:** 10.3390/ijerph15040643

**Published:** 2018-03-31

**Authors:** Alexandra Pitman, Adelia Khrisna Putri, Tanisha De Souza, Fiona Stevenson, Michael King, David Osborn, Nicola Morant

**Affiliations:** 1 UCL Division of Psychiatry, Maple House, 149 Tottenham Court Road, London W1T 7NF, UK; michael.king@ucl.ac.uk (M.K.); d.osborn@ucl.ac.uk (D.O.); n.morant@ucl.ac.uk (N.M.); 2 Camden and Islington NHS Foundation Trust, St Pancras Hospital, London NW1 0PE, UK; 3 UGM Faculty of Psychology, Universitas Gadjah Mada, Jl. Sosio Humaniora 1, Sleman, Yogyakarta 55281, Indonesia; adelia.k.p@ugm.ac.id; 4 North East London NHS Foundation Trust, Memory Service, Broad Street Health Centre, Morland Road, Dagenham, Essex, RM10 9HU, UK; Tanisha.DeSouza@nelft.nhs.uk; 5 UCL Research Department of Primary Care & Population Health, Rowland Hill St, London NW3 2PF, UK; f.stevenson@ucl.ac.uk

**Keywords:** suicide, bereavement, education, work, occupational functioning, qualitative research

## Abstract

People bereaved by suicide are at an increased risk of suicide and of dropping out of education or work. Explanations for these associations are unclear, and more research is needed to understand how improving support in educational or work settings for people bereaved by suicide might contribute to reducing suicide risk. Our objective was to explore the impact of suicide on occupational functioning. We conducted a cross-sectional online study of bereaved adults aged 18–40, recruited from staff and students of British higher educational institutions in 2010. We used thematic analysis to analyse free text responses to two questions probing the impact of suicide bereavement on work and education. Our analysis of responses from 460 adults bereaved by suicide identified three main themes: (i) specific aspects of grief that impacted on work performance, cognitive and emotional domains, and social confidence; (ii) structural challenges in work or educational settings including a lack of institutional support, the impact of taking time off, and changes to caring roles; and (iii) new perspectives on the role of work, including determination to achieve. Institutional support should be tailored to take account of the difficulties and experiences described.

## 1. Introduction

People bereaved by suicide have become a policy priority in the field of suicide prevention due to recent evidence identifying them as at an increased risk of suicide [1,2] and suicide attempt [2,3], as well as a greater public awareness of the devastating emotional impact of suicide loss. In order to develop effective suicide prevention interventions for this group and respond to their distress, researchers need to identify key factors in the pathway to suicide after suicide loss. Previous research has suggested explanations based on specific difficulties faced by the suicide-bereaved, including stigma [3,4], poor physical and mental health [2], suicide suggestion [5], and poor occupational functioning [6]. A recent finding is that people bereaved by suicide are significantly more likely than people bereaved by other causes of sudden death to drop out of a course or a job after their loss [6]. This warrants further investigation to understand the wider needs of this group, including any barriers to support. The current study adds to our understanding of these occupational impacts by exploring the nature of difficulties experienced in education or work after the suicide of a friend or relative.

Suicide bereavement is a frequent occurrence, even though the stigma of suicide leads many people not to mention it. The WHO estimates that 10 people are deeply affected by each suicide death, while efforts at empirical estimates suggest a figure of 60 [7] and more recently, 135 [8]. The lifetime prevalence of losing a close friend or relative to suicide is estimated at 22%, with 4% past-year prevalence [9]. In a US population sample, 51% knew one or more persons who had died by suicide, and 35% of these people experienced moderate to extreme emotional distress following the death, even up to 14 years later [10]. Given that one in 10 people from a UK sample of suicide-bereaved adults subsequently attempted suicide [6], it is important to understand the difficulties faced by a group prioritised in many international suicide prevention strategies. 

Poor occupational outcomes among people bereaved by suicide are of interest as candidate mediators of suicide risk because of the key roles of education in influencing future employment and income potential [11] and of work in influencing income, identity, and self-worth [12]. Socio-economic factors, as well as cognitive and emotional factors such as defeat, humiliation, and entrapment, feature prominently in theoretical models of suicidal behaviour [13]. Poor mental health in education is associated with dropout from vocational and higher educational courses [11]. In work settings, mental health problems cost the UK economy approximately £8 billion in absenteeism and £8 billion in staff turnover [14]. Adverse life events such as bereavement have also been shown to reduce productivity, with scores on a standardised task showing that people with experience of family bereavement or illness were significantly less happy and significantly less productive than those unexposed to such life events [15]. 

Studies specific to suicide loss show that, compared with the general population, suicide-bereaved adults are more likely to take extended periods of sick leave, to be unemployed, and to claim disability pensions [2]. Compared with people bereaved by other causes of sudden death, people bereaved by suicide are more likely have a low income [16] and to drop out of education or work [6]. Our national survey of people bereaved by suicide and other sudden deaths found that people bereaved by suicide were 80% more likely than people bereaved by sudden natural causes of death to drop out of a job or a course [6]. However, the same study found no group differences on depression, suggesting that factors other than mental illness impacted negatively on work or student roles. Indeed, the analysis suggested that higher levels of perceived stigma among the suicide-bereaved might account for the association with occupational drop-out [6]. This suggests that explanations may lie in a lack of support and confidence at work, in addition to difficulties in work relationships. A separate analysis found that people bereaved by suicide were significantly less likely to have received support from family, friends, and colleagues after their loss than people bereaved by other causes of sudden death, and were also more likely to report delays in receiving support [17]. 

Our qualitative work investigating the stigma perceived by people bereaved suddenly found that friends and colleagues tend to avoid the bereaved, failing to offer support [4]. This was particularly extreme for people bereaved by suicide, who understood this as being driven by others not knowing what to say. This social avoidance was experienced as stigmatising, and also provoked resentment [4]. A survey of support group attendees bereaved by a child’s suicide inquired about relationships with colleagues after the suicide, and found that 17% reported hurtful responses from colleagues and 22% reported strained relationships with colleagues after the loss, but 40% reported helpful responses from colleagues [18]. 

Little other research has investigated occupational functioning specifically after sudden bereavement, tending instead to include bereavements by any cause. There is a need for further understanding of the difficulties faced by people bereaved by suicide in occupational settings, as specific targeted interventions in the workplace or educational institutions have the potential to mitigate their risk of suicide. Our aim was to elicit the views of a national sample of young adults bereaved by the suicide of a close friend or relative on whether and how the bereavement had affected their educational or work performance. In analyzing their responses, our objective was to identify key themes in their experiences, whether positive or negative, and infer from this how occupational support might be improved.

## 2. Materials and Methods 

### 2.1. Study Design and Participants

We conducted a national cross-sectional study of young bereaved adults in the United Kingdom in 2010; the UCL Bereavement Study. The primary objective of the larger online survey was to investigate the association between bereavement by suicide and adverse mental health and social outcomes. Our findings supporting an association with risk of suicide attempt, poor occupational functioning, poor social functioning, and perceived stigma are reported elsewhere [3,6]. The survey also collected qualitative data in order to answer research questions about the impact of the bereavement on social and occupational functioning, with the aim of analysing these data once the quantitative analyses were complete. 

As described previously [6], recruitment focussed on young adults as this was the age group of greatest policy interest in terms of suicide risk at the time of designing the study [19]. Our inclusion criteria were: adults aged 18–40 who, since the age of ten, had experienced sudden bereavement of a close friend or relative. Our upper age limit was higher than that of 24 used by the WHO for young adults [20] to avoid collecting only recent experiences of bereaved adults. We excluded those exposed to sudden bereavement in childhood to reduce recall bias and capture experiences above the threshold for adult cognition. We sampled via the email systems of large higher education institutions (HEIs) in the UK, in order to access hard-to-reach groups, especially men. Institutions (listed in full in the acknowledgements) included veterinary, agricultural, art, and drama colleges, thus offering access to a large, varied but defined sample of young adults. This approach avoided the biases associated with recruiting from bereavement support groups and accessing only a help-seeking sample [21]. 

Of all 164 UK HEIs invited to participate in our study, 37 (23%) agreed to take part, with a higher response (40%) from the group of universities characterised by a higher research income. This provided direct access to a sampling frame of 659,572 staff and students, each of whom received an individual email inviting them to take part in a survey of “the impact of sudden bereavement on young adults”, including an embedded survey link. Ten HEIs varied this approach by emailing students only, including the invitation within their weekly newsletter email, or advertising it on their intranet. 

### 2.2. Procedures

An on-line questionnaire was designed by AP, FS, DO, and MK [6] in consultation with a group of young bereaved adults and bereavement counsellors, who advised on wording and which domains to cover. We piloted the questionnaire nationally, as described elsewhere [6]. 

The online survey included 119 fixed-response questions, to collect quantitative data on socio-demographic and clinical characteristics. Part 2 of the questionnaire contained 20 open questions probing specific dimensions of experiences of bereavement. There was no upper word limit on free text responses. Respondents were invited to give as much or little detail as they wished, or to skip the question. Two of these questions explored the impact of the bereavement on education and work performance, and were worded as follows:

“*In what way, if any, has the bereavement affected your educational progress?*”

“*What about your work performance?*”

### 2.3. Ethics

Online participant information indicated that the UCL research team responsible for the study included psychiatrists (AP, DO, MK) and a medical sociologist (FS), and that all data would be anonymised and handled as per data protection legislation. All participants provided online informed consent. The study protocol was approved by the UCL Research Ethics Committee in 2010 (reference: 1975/002). 

### 2.4. Analytic Approach

We used an adapted form of thematic analysis [22] to analyse free text responses to these two questions, restricting our analysis to respondents bereaved by suicide. We took an inductive exploratory approach, and conducted analysis collaboratively as a cross-disciplinary team comprising perspectives from psychology, sociology, and psychiatry. This encouraged personal reflexivity by challenging differences in interpretation and reducing the influence of theoretical or personal conceptions [23].

Data were imported into Microsoft Excel, which was used to support organising, reviewing, and coding large volumes of linked qualitative responses. After data familiarization, we coded responses using a basic five-part classification for responses to questions about the impact of the bereavement: negative, neutral (primarily “none” or “N/A”), positive, both positive and negative, and ambiguous. 

We then moved to a more content-based, fine-grained approach. Neutral responses that provided no insights (for example “*none at all*”), and ambiguous responses, (for example “*I’m not sure*” or “*Bereavement occurred two weeks before A level exams*”) which tended to be brief, were excluded from this analysis. Following initial discussions about emergent codes, two members of the team (AKP and TDS) independently coded a randomly selected sub-sample of 100 responses, and compared their work for consistency. The larger analytic team then reviewed and discussed the initial coding framework, resolving discrepancies and amending the emergent framework. Following general coding agreement at this stage, AKP continued to code the full dataset, using regular discussions within the research team to clarify the conceptual meanings of thematic codes as coding progressed. Through further team discussions, we built up a framework of new codes, sub-codes, collapsed codes, and higher-order categories. We then reviewed the revised framework to explore the distribution of responses across codes, and in relation to higher-order themes as a final validation of the conceptual coherence of the analysis. We used COREQ guidelines to structure the reporting of our findings [24]. 

We used Stata version 12 (StataCorp., College Station, TX, USA) to present descriptive characteristics for the qualitative sample, compared with the wider quantitative sample of suicide-bereaved adults.

The dataset generated and analysed in the current study is not publicly available due to the risk of identifying participants. Requests to analyse these data should be made to the corresponding author, subject to honorary contract status approval in the UCL Division of Psychiatry. 

## 3. Results

### 3.1. Sample Characteristics 

Of the estimated 659,572 bereaved and non-bereaved people receiving the email invitation, 5085 people responded to the questionnaire by clicking on the survey link, and 4630 (91%) consented to participate in the online study. An overall response rate could not be accurately measured, as the precise denominator of people bereaved by suicide in the UK is not obtainable using routine data or survey methods. A total of 614 people reported having been bereaved by suicide and provided responses to the quantitative component of the questionnaire. Of these, 460 (75%) completed at least one of the two questions probing the impact of bereavement on educational or occupational functioning. 

Our qualitative sample was predominantly female (81%) and of white ethnicity (92%). As per inclusion criteria, respondents were aged between 18 and 40, with a median age of 23 (see Table 1). Median age at the time of the bereavement was 19 (interquartile range (IQR) = 17–23), and median number of years since the loss was three (IQR = 1–7). The sample was equally split between relatives (51%) and non-relatives (49%) of the deceased. In the majority of cases (72%), the deceased had been male and the median age of the deceased was 27 (IQR = 20–45). The bereavement had occurred between three days and 26 years prior to filling in the questionnaire, with a median value of three years (IQR = 1–7). These characteristics matched those of the wider sample of 614 suicide-bereaved respondents (Table 1).

### 3.2. Qualitative Findings

Our initial basic classification of responses is presented in Table 2, with negative impacts reported most commonly for both educational (50%) and work (36%) contexts. 

Following the exclusion of very brief responses (<10 characters) and ambiguous responses, we excluded 31 respondents who gave very brief responses on both questions and one respondent who gave ambiguous responses (“*very little*”) on both. We conducted our more in-depth thematic analysis of extensive responses from 428 survey respondents, and identified three higher-order themes, each with sub-themes. These are summarised in Table 3 and described in more detail below. Illustrative quotes are presented as typed into the online questionnaire, and only amended where correcting spelling errors. In the UK GCSEs are public exams taken at age 16, and A levels are those taken at 18. The term ‘uni’ denotes the abbreviation of university. 

#### 3.2.1. Specific Aspects of Grief Diminishing Work Performance

It was common for specific components of grief following the suicide to be cited as reasons for falling behind with work or producing work of a poor quality. Coping with tearfulness, anger, profound sadness, poor concentration, confusion, or anxiety was a struggle for many, experienced as frustrating and sometimes embarrassing.

##### Cognitive Aspects of Grief

Contrary to our expectations, cognitive aspects of grief were more prominent than emotional aspects of grief, and it was common for respondents to describe problems with concentration and motivation affecting their productivity and drive. A sense of futility was often described, as part of a change in world view, which resulted in a lack of interest or drive. These problems sometimes emerged or persisted for some time after the bereavement.

“*I’ve lost all focus and drive. I no longer want to be at university, I feel it’s futile….I’ve gone from being a high achiever, always busy, doing well to not doing anything. I have no focus, I can stare at the same page, not being able to read it and suddenly the entire day has gone by without me realising. Even when I manage to write something it’s not very good because my brain’s not firing in the way it used to and isn’t making all the proper connections to write good work*.”

(Male, 21 years old, in part-time paid work, bereaved less than a year previously by the suicide of his partner)

Motivation was such a problem for some people that their grades fell, or they dropped out of school, college, or university. This resulted in what were perceived to be significant departures from the trajectory they might otherwise have achieved.

“*A suicide changes how you see the world, especially if you are relatively young and not used to serious turmoil. This obviously has an effect on how positive a person can remain, or whether positivity is even a reasonable consideration. I still study, but my aims have changed. I only study in the hope that I will make money out of what I can learn. I have no real interest in what I am doing anymore, and I don’t find inspiration in many things*.”

(Male, 30 years old, no information on student/staff status, bereaved two years previously by the suicide of a close friend)

##### Emotional Aspects of Grief

Managing emotions at work was an additional struggle for some respondents, and crying in front of colleagues or clients was described as particularly embarrassing. Others described having become more sensitive to criticism or negative feedback, resulting in thoughts and sometimes expressions of anger. These emotional outbursts could make working in a team very challenging. Managing a more general anger at the world was also linked to low motivation, and was cited as a reason for dropping out of jobs or courses.

“*I was at University at the time when it happened and I dropped out. I just became angry with the world and drank a lot in the evenings*.”

(Female, 36 years old, no information on student/staff status, bereaved 12 years previously by the suicide of her father)

“*I would cry at work which in return caused me to feel like I had to apologise to everyone for making them feel a bit uncomfortable*.”

(Female, 25 years old, Full-time student with part-time job, bereaved over three years previously by the suicide of her uncle)

Significant anxiety was described by many, whether in relation to specific triggers or a general state of low mood and anxiety. The impact of anxiety on work performance may have not been sufficiently taken into account by managers or teaching staff, and respondents sometimes reported a lack of understanding, resulting in them being sanctioned for poor attendance.

“*It was in the middle of my year 2 exams. I have done badly in all of them and I am worried about not reaching 3rd year. When I look at my notes to revise I am reminded constantly of my brother. I can’t revise and I get anxious and uncomfortable. I then worry about the exam and I get worse. I fear for my education*.”

(Male, 20 years old, full-time student, bereaved one month previously by the suicide of his brother)

“*I rarely attended sixth form and often took time off uni when I was anxious/depressed although they rarely understood and I was punished for attendance several times*”

(Female, 21 years old, full-time student, bereaved four years previously by the suicide of her father)

Many described the emergence (or recurrence) of mental health problems as attributable to the stress of managing extreme grief after the suicide, affectingtheir occupational and social roles.

“*I was in my last year at University when the bereavement occurred and became extremely stressed and depressed. It meant that trying to complete my dissertation project and prepare other coursework became extremely difficult. I was supported by my partner though and was able to complete my degree satisfactorily (at one point I had contemplated dropping out)*.”

(Female, 26 years old, in full-time paid work, bereaved four years previously by the suicide of her uncle)

“*…my cousin’s suicide was a factor in me developing anorexia. I believe it was partly, especially because I took on too many problems and weigh myself down. As a result of my illness I missed a year of school and didn’t take any GCSEs. I also had to take a break from university*.”

(Female, 22 years old, Full-time student, bereaved 10 years previously by the suicide of her cousin)

These mental health effects were not always apparent immediately. Some respondents described putting their feelings ‘in a box’ in order to get on with work or studying, but then breaking down subsequently. Where mental health problems surfaced much later, it seemed hard for employers or teaching staff to link them to the suicide bereavement, even if a clear association was perceived by the bereaved.

“*the combination of putting my feelings into a box for a year and starting uni did not combine well and caused me to break down a bit and the box opened with fairly disastrous consequences on my happiness for a period and this affected my concentration at uni. Many times I couldn’t raise the energy to get up to go to lectures or when I was there I was so stressed and upset that I couldn’t see the slides properly as I was so anxious.*”

(Male, 21 years old, full-time student with part-time job, bereaved three years previously by the suicide of a close friend)

##### Impact of Grief on Social Confidence

Many described a loss of self-confidence after the bereavement, becoming more socially withdrawn and finding it harder to work in team or client-facing environments. This seemed to be linked to many of the cognitive and emotional aspects of bereavement described above, such as breaking down when talking about the death, generally crying more, becoming more sensitive or depressed, or losing interest in one’s work. This theme captured how debilitating the cognitive and emotional aspects of grief after a suicide were for someone trying to maintain professionalism or meet deadlines.

“*At the time my teaching suffered, I was less communicative with my colleagues and cared little about what I was doing*.”

(Female, 27 years old, full-time student, bereaved three years previously by the suicide of her ex-partner)

“*The death has made me lack confidence more than I did, I do not believe in my capabilities in the work-place and often get stressed in tricky situations with customers (I work in retail). I also feel less able to relate to and interact with my work colleagues*.”

(Female, 23 years old, full-time student with part-time job, bereaved two years previously by suicide of her father)

Whilst the disabling effects of grief described above might apply to any bereavement, one specific aspect of suicide bereavement eroded confidence: the fear of being stigmatised by the suicide. Respondents often chose to remain vague about the cause of death, or avoid the subject. This side-stepping of ‘small talk’ came at the cost of appearing unfriendly, but this was usually felt to be preferable to being judged, ostracised, or avoided. However, these restraints on social interactions also affected social confidence by creating awkwardness in social situations at work. This was a particular problem in roles where making conversation was an important part of customer or team relationships.

“*I found my low confidence extremely limiting in my interaction with my colleagues… I couldn’t bring myself to talk to them and much preferred to be left alone. The only problem was that naturally I was thus perceived as aloof, as I certainly wasn’t about to risk entrusting my boss or colleagues with the horrific information about my bereavement. I was (and still am) convinced that I would be labelled as nuts because such a thing transpired in my family, even though I had no part in it whatsoever*.”

(Female, 31 years old, full-time student, bereaved over six years previously by the suicide of her brother)

“*As a hairdresser I couldn’t talk to people, so regulars wanted to ask me and I hated them for it, and when a client didn’t know I was annoyed they seemed heartless. Had to quit working due to emotional distress, bad work performance, also no motivation*.”

(Female, 33 years old, no information on student/staff status, bereaved nine years previously by the suicide of her partner)

#### 3.2.2. Structural Challenges in Work or Educational Settings 

Some respondents described external factors that had hampered their ability to cope with work pressures whilst grieving the loss. These often related to a lack of support, or the shouldering of additional burdens. 

##### Lack of Institutional Support

Many respondents were appalled at the lack of institutional support offered or available, and provided examples of the insensitivity of managers or teaching staff. Such experiences clearly exacerbated difficulties and were experienced as setbacks in their own right. Examples included encountering workplace or university regulations that appeared insensitive, such as eligibility for compassionate leave extending only to close relatives or being contingent on providing a copy of the death certificate. Dealing with an inflexible system was a problem for both bereaved relatives and friends, particularly where it appeared to call into question the validity of their relationship to the deceased and that person’s need to grieve.

“*The reaction from the school’s administrative and counselling staff left (me) first horrified and then disillusioned. I barely graduated high school*.”

(Female, 38 years old, full-time student with part-time job, bereaved 24 years previously by the suicide of a friend)

“*had a lot of issues with work, don’t feel they suitably appreciated the effect and length of effect on me, I was disciplined a lot for sickness absence, and no account was taken that emotional issues could be affecting my physical health*”

(Female, 31 years old, part-time student with part-time job, bereaved three years previously by the suicide of her brother)

Such responses lay in contrast to the one response (coded as neutral) in which the support of colleagues was presented as a factor buffering the potential negative effects of the bereavement.

“*Did not affect my work performance. My colleagues were all very supportive.*”

(Female, 34 years old, in full-time paid work, bereaved two years previously by the suicide of a close friend)

##### Impact of Taking Time Out

For those who were entitled to take compassionate leave, primarily relatives, this was valued in providing time to grieve. However, returning to a backlog of work, particularly with impaired concentration, was very difficult. The pressure to catch up with tasks whilst continuing to grieve contributed to stress. Tasks or assignments were often described as having been completed in a rush, impairing the quality of the work. Others felt their confidence in their abilities at work had diminished in their absence.

“*My university provided work placements, I had to make up the 5 weeks which I had missed immediately following my brother’s death …I tried to work full-time alongside studying for my final exam, and became very depressed, anxious, tearful and stressed*.”

(Female, 24 years old, no information on student/staff status, bereaved one year previously by the suicide of her brother)

Taking long-term unpaid leave also gave rise to financial problems for several respondents. There was therefore a layering effect of bereavement, work stress, and financial instability.

“*Had to take time off college which brought my attendance down which affected my allowance making it difficult to concentrate on learning as it created money problems*.”

(Female, 18 years old, Full time student with part-time job, bereaved over one year previously by the suicide of her father)

##### Changes in Caring Roles

A further structural challenge faced by many respondents, particularly relatives, was the loss of emotional support previously provided by the deceased. Often this related to the death of a parent or to a change in the relationship with a grieving relative. Losing this support meant losing someone who had normally encouraged the respondent to pursue their studies, or had looked after the home and family to facilitate work and student roles. For some it meant taking on a caring role within the family, with the associated costs of time, energy, and emotion. In a few cases it meant quitting jobs or courses to live nearer the family or to earn more to support them. These sacrifices, although felt to be the right decision, did significantly disrupt progress in work or education.

“*My school work suffered at the time, both because my home life was disrupted and also because my mother, who was suffering the most, was the person who would check up on me, my homework etc...She was totally absent during this time*.”

(Female, 25 years old, full-time student with part-time job, bereaved 16 years previously by the suicide of an uncle)

“*my sister was still extremely traumatized by the incident and I found it very difficult to refuse her if she expressed an interest in life at all really, because I knew that my being there was helping her get her own life back on track…it was clear to me that I was helping her to recover. Therefore, my usual 10 hours of revision a day went down the drain and I ultimately got 58% in the exams that year, when I know I should’ve got a lot more*.”

(Female, 21 years old, full-time student, bereaved over two years previously by the suicide of her uncle by marriage)

#### 3.2.3. New Perspectives on the Role of Work

A small sub-group of respondents, who tended to have been bereaved some years previously, described positive impacts on their work output. This was primarily due to new motivation and drive born out of the loss, and a sense that the suicide had been life-changing in forcing them to re-evaluate much in their lives. Although this minority of respondents were reflecting back over some years, their responses conveyed that this motivation had set in from the start, as a way of coping with the immediate aftermath of the death. 

##### Work as Distraction or Escape from Emotions

For many of this group, work offered distraction from difficult emotions and traumatic memories. They described focussing energies on exam revision, a degree course, or projects at work, finding the structure and familiarity of work containing. Usually, it was reported that performance had improved due to the extra hours and attention devoted to work.

“*I think for me, because I liked to study and generally liked being at school, when my mother died this became a kind of support for me. I threw everything into my studies; it was what I knew best, it was a distraction too. This continued through college, university and is probably a reason why I am now a lecturer at a university—it’s almost like the education system has offered structure, meaning and support to me; these were lacking at home after my mother died*.”

(34 year old woman, in full-time paid work, bereaved 22 years previously by the suicide of her mother)

“*I was doing a FE diploma at the time. I coped with it as it was structured and time-consuming and offered me a distraction*.”

(Female, 27 years old, full-time student, bereaved three years previously by the suicide of an ex-partner)

These respondents often described using the distraction of work as an escape from dealing with their grief, primarily by blocking out feelings.

“*I worked very hard on my finals to block out thoughts of her death. I felt a bit guilty for this at the time, but knew she wouldn’t want us all to fail. I therefore put everything into my academic work as I have always done*.”

(Female, 36 years old, full-time student, bereaved 15 years previously by the suicide of a close friend)

“*I went numb and focussed purely on my degree, and studies I think in a way to block out the traumatic events.*”

(Female, 29 years old, full-time student with part-time job, bereaved seven years previously by the suicide of her sister)

##### Motivation to Honour the Deceased

Increased motivation to succeed was often described in relation to honouring the deceased and finding a way to make them proud. Choices in work and education were sometimes made with this influence in mind. For some, this meant following an educational or career path that the deceased would have approved of, or pursuing a specific goal that emulated the achievements and positive traits of the deceased. This was often cited as a motivation for entering higher education. Responses conveyed a sense of the deceased being a powerful but invisible role model, providing comfort through an imagined sense of them approving these life choices or achievements.

“*I felt apathetic towards education initially after the bereavement, but then applied myself to work as my friend valued education very highly, as do I normally. He wanted to be a human rights lawyer*.”

(Female, 20 years old, in part-time paid work, bereaved seven years previously by the suicide of a close friend)

“*I think it has made me determined to complete my degree so that my dad is proud of me.*”

(Female, 25 years old, in full-time paid work, bereaved three years previously by the suicide of her father)

##### Motivation to Live Life to the Full 

Motivation to succeed at work was sometimes described more generally as part of a desire to live life to its full possibilities. Losing someone in traumatic circumstances had prompted some respondents to reflect on their life so far, giving them a determination to take more control of their choices or revise past poor decision-making.

“*The bereavement has given me the drive to pursue my education. It made me realise that life is too short to pass opportunities and be unhappy in situations. If I had not suffered the bereavement I may have never gone back to higher education*.”

(Female, 30 years old, full-time student, bereaved six years previously by the suicide of her partner)

“*It spurs me on to perform better—we only live once—I want to make the most of it. However I have a more healthy philosophy about my work/life balance and avoid getting too stressed out anymore—I constantly remind myself about what is really important*.”

(Female, 25 years old, full-time student with part-time job, bereaved five months previously by the by suicide of a cousin)

##### Motivation to Join Caring Professions

The experience of a loved one’s suicide often motivated an interest in careers related to mental health or the care of vulnerable individuals, sometimes prompting a complete career change. This was largely explained by the development of greater empathy for those in distress and a drive to prevent more people from making a suicide attempt.

“*It made me want to make something of myself and has made me decide to want to help people with mental health problems*.”

(Female, 22 years old, full-time student, bereaved three years previously by the suicide of her father)

“*It changed how I view life..…I left a well-paid career on a career break and did not return, choosing instead to work with vulnerable young people and adults for 2/3 of my previous salary*.”

(Female, 36 years old, full-time student with part-time job, bereaved 10 years previously by the suicide of her partner)

Those who worked in roles with caring or pastoral responsibilities reported having gained more empathy from their experience of suicide, meaning they were able to provide better care and support for those they worked with.

“*I have always wanted to nurse, and after my husband died it spurred me on to do my degree and follow my dreams for me and for my family.….it’s changed me a great deal, maybe because I can empathise a lot more with relatives and patients in a very different way, I am a harder worker now than I ever was!*”

(32 year old female, full-time student, bereaved 11 years previously by the suicide of her partner)

## 4. Discussion

### 4.1. Main Findings

Our study provides qualitative evidence that suicide bereavement takes a significant toll on academic and workplace achievements in a primarily student sample. Specific features of grief, experienced intensely, were seen as particularly detrimental to making progress in coursework, exam revision, or work tasks, and in communicating with colleagues. The pressures of lagging behind through time taken off or additional responsibilities added to work stresses. Our analysis also identified weaknesses in the institutional support on offer to students and employees bereaved traumatically, and clear indications as to how these might be improved. A small minority described a positive impact of the suicide in providing them with greater motivation and drive. 

This study adds the necessary context to our previous findings of a greater probability of drop-out from work or education in people bereaved by suicide [6]. It is clear that specific aspects of grief, such as tearfulness, anger, reduced motivation, poor concentration, and anxiety, have a major impact on educational and work performance. Loss of motivation is likely to influence a decision to drop out of a course or job. Severe emotional distress, including the emergence or recurrence of mental health problems, risks interrupting or ending a planned trajectory, despite best ambitions. Having to move home and lacking appropriate support are also factors likely to explain higher drop-out rates. 

Experiences of stigma were not as prominent as we had expected, beyond the observation that colleagues were avoidant in offering support and that social interactions were sometimes awkward. We observed some variation in themes by time since loss, such that those bereaved for longer tended to be those who described positive effects. This may have been a perspective that had developed over time, but perhaps was more subject to recall bias. We observed some variation by kinship, primarily in relation to changes in caring roles within the family structure. Those who had lost a close friend appeared to be as affected by emotional, cognitive, and social aspects of grief as those who had lost a relative or friend. 

### 4.2. Results in the Context of Other Studies

No other qualitative studies have investigated occupational functioning specifically after suicide bereavement. However, our findings match those of a recent Australia qualitative study of adolescents bereaved by suicide and other deaths, in which alongside features of disabling grief, bereaved adolescents described the death as a catalyst for personal growth [25]. This marked the start of lasting positive changes in their perceptions of self, relationships, or life [25]. Similarly, in a US sample of college students bereaved by a parent’s death by any cause, analysis of interview data suggested that they too were able to use their loss as a catalyst for personal growth and increased motivation [26]. Furthermore, in the quantitative component of that study, the greater the subjects’ ability to find meaning and make sense of the loss, the less intense was their grief [26]. 

The disabling cognitive and emotional aspects of grief described in our study mirror the wider bereavement literature. Moderate disruptions in functioning are understood to be normal in the first year after any loss, including difficulties adapting to occupational roles and dissatisfaction over work performance [27]. Initial numbing, impaired judgment, and concentration [28] may become more chronic in the minority who develop complicated grief [27]. The social isolation and lack of support we described is also consistent with empirical studies of bereavement describing social relationships, including work relationships [29,30,31]. Lack of institutional support is also common to other bereaved groups. A British survey for the National Council for Palliative Care [32] found that 32% of recently bereaved people in work at the time said they were not treated with compassion by their employer [33]. 

### 4.3. Strengths and Limitations

To our knowledge, this is the first study to investigate the impact of suicide loss on educational and occupational functioning using qualitative methods. It complements the findings of our quantitative work, by exploring the reasons underlying the association between suicide bereavement and occupational dropout. Although we did not compare qualitative data from respondents bereaved by other causes of death, we assume that in bringing together these quantitative and qualitative findings we have been able to explore why the quantitative association with occupational difficulties is so pronounced for people bereaved by suicide. However, without directly comparing the qualitative accounts of the suicide-bereaved with those bereaved by other causes, we cannot assume that our findings apply solely to the former. As our study lacked a temporal dimension, it was unclear how long initial problems persisted for. Our study also lacked any diagnostic instruments, and we remain unclear as to whether the cognitive and emotional features described reached thresholds for psychiatric disorder.

We accessed a large sample of UK-based staff and students in HEIs, and a wide range of views, whilst avoiding a help-seeking sample. However, our sampling method resulted in over-representation of white individuals and highly-educated females. Restriction of sampling to students only in some institutions may have biased recruitment to a younger-aged sample, and 78% of our sample constituted current students. Many of the staff we sampled described the impact of a suicide loss experienced whilst still a student, so our findings may be more representative of the impact of suicide on students rather than those in work. As 99% of our sample was in education or working in education, this group might be viewed as more motivated by academic achievement than other groups, or valuing this more. Surveys from samples more representative of the working and unemployed population would complement our findings. Our internet-mediated approach reduced the effects of interviewer presence, particularly social desirability effects [21], and allowed us to collect large volumes of data. However, a response bias is possible from those who had been most adversely affected in their work or studies, with quantitative data from our wider sample of suicide-bereaved respondents indicating that 8% had dropped out of work or education after their loss [6]. Our use of open questions allowed respondents freedom to identify issues of importance to them. However, responding online meant we were unable to probe key points in more detail, or respond if participants became distressed. This meant that we sometimes lacked information needed to interpret responses in the context of respondents’ wider positions and culture. 

Our collaborative data analytic approach helped us reduce the influence of theoretical or personal pre-conceptions by challenging differences in interpretation. Given cultural dimensions to attitudes towards suicide in educational and work settings, the age range sampled, the high proportion of white respondents and students, and the possibility of selection bias (favouring higher social classes) and male non-response bias, the results of this study may only be generalisable to young bereaved women, white ethnic groups, students, and the highly educated. Nevertheless, as the only qualitative study of occupational functioning after suicide loss, it represents the best available evidence.

### 4.4. Clinical and Policy Implications

Postvention is the term used to describe suicide prevention interventions delivered after a suicide, with the intention of prevention further suicides. Schools, colleges, and universities have a duty to safeguard the mental health of their students, and the workplace is now seen as a setting for mental health promotion [34]. Postvention guidelines have been developed for schools [35], and employers [36,37,38,39], explaining how to support a colleague bereaved by suicide, and our findings suggest that these should be widely disseminated. Future editions should highlight our finding that occupational difficulties after bereavement are not limited to emotional aspects, but also include problems with concentration and motivation. Staff should be trained to view any deficits in performance in the context of the impacts described here, set deadlines considerately, and familiarise themselves with bereavement support services. Restrictive compassionate leave policies should take into account our findings, and evidence that perceptions of closeness to the deceased predict the likelihood of developing depression or anxiety after the loss [40,41]. These present a rationale for widening such rigid eligibility. Finally, as workplace culture varies internationally and by industry, there is a need for postvention guides on a country-by-country and industry basis. 

### 4.5. Future Research

Future research should investigate the impact of suicide bereavement on occupational functioning in other countries, and compare functioning after suicide to that after other losses. There is also a need to explore responses to suicide loss in minority ethnic groups, particularly given findings that bereaved people from non-white groups report hiding their emotions and concealing their loss [25]. Longitudinal research on occupational functioning after suicide would also identify how productivity varies over time, identifying optimal points for intervention. Qualitative work on needs for support within educational and workplace settings would also help in the design of systems of support. 

## 5. Conclusions

We identified a very negative picture of the impact of suicide loss on occupational functioning. Respondents to our national survey described their struggles with concentration, motivation, tearfulness, anxiety, and confidence, in addition to difficulties with inter-personal dynamics, time pressures, and a lack of support. However, a minority described the suicide loss as a landmark in finding a new determination to achieve, sometimes by providing distraction during a difficult period, and this sub-group found their work performance improving subsequently. Our work identifies a clear need to improve the support structures in place in schools, colleges, universities, and workplaces; to revise company policy or university regulations on leave and mitigating circumstances; and to train staff in how to make necessary allowances for the identified needs. Guidance on how colleagues can support someone bereaved by suicide would also help foster supportive peer relationships and assist the suicide-bereaved in their return to work or educational settings. 

## Figures and Tables

**Table 1:** Demographic characteristics of the 460 respondents compared to the wider sample of 614 suicide-bereaved respondents.

Characteristic	*n* = 614 Suicide-Bereaved Respondents	*n* = 460 Suicide-Bereaved Respondents in Qualitative Sample
gender		
male *n* (%)	115 (19)	76 (17)
female *n* (%)	499 (81)	384 (83)
median age (IQR)	23 (20–29)	23 (21–29)
age group		
aged 18–21 *n* (%)	224 (36)	163 (35)
aged 22–40 *n* (%)	390 (64)	297 (65)
work status		
working in paid job *n* (%)	68 (11)	100 (22)
studying at college/university *n* (%)	526 (86)	211 (46)
both working and studying *n* (%)	20 (3)	146 (32)
neither (on leave/unemployed) *n* (%)	0 (0)	3 (1)
educational attainment		
educated to maximum A level *n* (%)	255 (42)	183 (40)
educated to degree level or above *n* (%)	359 (59)	277 (60)
socio-economic status ^¥^		
social classes 1 and 2 *n* (%)	380 (64)	280 (61)
social classes 3 to 7 & 9 *n* (%)	213 (35)	170 (37)
missing *n* (%)	21 (3)	10 (2)
ethnicity		
white *n* (%)	562 (92)	423 (92)
Asian/Asian British *n* (%)	15 (2)	11 (2)
Black/Black British *n* (%)	9 (1)	6 (1)
Mixed race *n* (%)	20 (3)	14 (3)
Other *n* (%)	8 (1)	6 (1)
number of sick days in last year		
0–7 days sick *n* (%)	337 (55)	256 (56)
8–365 days sick *n* (%)	153 (25)	118 (26)
Missing *n* (%)	124 (20)	86 (19)
median number of sick days in last year (IQR)	4 (1–10)	5 (1–10)
median age bereaved (IQR)	19 (17–23)	19 (17–23)
median time since bereavement, years (IQR)	3 (1–7)	3 (1–7)
gender of the deceased		
male *n* (%)	433 (71)	331 (72)
female *n* (%)	181 (29)	129 (28)
median age of the deceased (IQR)	19 (17–23)	27 (20–45)
kinship		
father *n* (%)	86 (14)	73 (16)
mother *n* (%)	31 (5)	25 (5)
brother (including half-siblings) *n* (%)	46 (7)	38 (8)
sister (including half-siblings) *n* (%)	15 (2)	11 (3)
grandparent *n* (%)	11 (2)	9 (2)
uncle/aunt *n* (%)	49 (8)	33 (7)
niece/nephew *n* (%)	4 (<1)	3 (1)
cousin *n* (%)	54 (9)	42 (9)
close friend (including colleagues) *n* (%)	250 (41)	166 (36)
partner/spouse *n* (%)	23 (4)	22 (5)
ex-partner/ex-spouse *n* (%)	15 (2)	13 (3)
in law/step/adoptive relation *n* (%)	23 (4)	19 (4)
missing *n* (%) *	7 (1)	6 (1)

Key: ^¥^ using the National Statistics Socio-economic Classification (NS-SEC) based on the Office for National Statistics Standard Occupational Classification 2010 (SOC2010). * all missing cases for this variable were identified as non-relatives apart from one who did not indicate kinship. IQR = interquartile range; SD = standard deviation.

**Table 2:** Basic classification of 460 responses.

Category	Educational Functioning *n* (%)	Work Functioning *n* (%)
negative	232 (50.0)	167 (36.3)
neutral	93 (20.2)	115 (25.0)
positive	66 (14.3)	39 (8.5)
both positive and negative	26 (5.6)	21 (4.6)
ambiguous	13 (2.8)	15 (3.3)
blank (for 1 of the 2 questions)	30 (6.9)	102 (22.2)
Total	460 (100)	460 (100)

**Table 3:** Descriptive themes.

Specific aspects of grief diminishing work performance	Cognitive aspects of grief Emotional aspects of grief Impact of grief on social confidence
Structural challenges	Lack of institutional support Impact of taking time out Changes in caring roles
New perspectives on the role of work	Work as distraction or escape from emotions Motivation to honour deceased Motivation to live life to full Motivation to join caring professions
